# Cytomegalovirus immune reconstitution inflammatory syndrome manifesting as acute appendicitis in an HIV-infected patient

**DOI:** 10.1186/1471-2334-14-313

**Published:** 2014-06-09

**Authors:** Kimberly F Faldetta, Sarah Kattakuzhy, Hao-Wei Wang, Irini Sereti, Virginia Sheikh

**Affiliations:** 1 Laboratory of Immunoregulation, National Institute of Allergy and Infectious Diseases, National Institutes of Health, Bethesda, MD, USA; 2 Laboratory of Immunoregulation, Leidos Biomedical Research, Inc, Frederick National Laboratory for Cancer Research, Frederick, MD 21702, USA; 3 Laboratory of Pathology, National Cancer Institute, National Institutes of Health, Bethesda, MD, USA

**Keywords:** Cytomegalovirus, Immune reconstitution inflammatory syndrome, Opportunistic infections, HIV

## Abstract

**Background:**

Appendicitis occurs with increased frequency in HIV infected compared to HIV uninfected persons. CMV-related appendicitis specifically presents with typical appendicitis symptoms including surgical abdomen, fever and leukocytosis and may have a more severe course with higher mortality than other types of infective appendicitis. We report the first case of CMV appendicitis as a manifestation of Immune Reconstitution Inflammatory Syndrome (IRIS).

**Case presentation:**

The patient was a 38 year old woman with a recent diagnosis of HIV infection who complained of right lower quadrant pain, anorexia, nausea and fevers two weeks after initiating antiretroviral therapy. Acute appendicitis was suspected and the patient underwent an appendectomy. Pathologic examination of the resected appendiceal tissue demonstrated inflammation with perforation and cytopathic changes typical of CMV that were positive for CMV by immunostain. This presentation of CMV abruptly after antiretroviral therapy initiation with a pronounced cellular infiltration of the tissue, is consistent with CMV-IRIS presenting as appendicitis.

**Conclusions:**

Appendicitis can be a rare manifestation of CMV-IRIS in HIV-infected patients who start antiretroviral therapy. Evaluation of appendiceal tissue for cytopathic changes and CMV should be considered in acute appendicitis in HIV infected persons.

## Background

Combination anti-retroviral therapy (ART) has reduced the mortality from cytomegalovirus (CMV) opportunistic infections in HIV positive patients [1]. CMV continues to result in morbidity and mortality in patients initiating ART at low CD4 T-cell counts, occasionally as a result of immune reconstitution inflammatory syndrome (IRIS). We present a case of unmasking CMV-IRIS in an HIV-infected patient following ART initiation.

Appendicitis occurs with increased frequency in HIV infected patients as compared to HIV uninfected patients [2]. A number of opportunistic pathogens or AIDS related malignancies have been shown to cause appendicitis in HIV-infected patients, including Kaposi sarcoma [3], Strongyloides [4], Mycobacterium tuberculosis [5], and Mycobacterium avium complex [6].

Tucker et al. published the first report of CMV appendicitis in an HIV positive patient in 1989 [7] and, since that time, ten additional reports of CMV appendicitis in HIV patients have been described in the literature [8-16]. Ten of the eleven reported patients presented with right lower quadrant pain and in eight of them fever was noted at the time of presentation (see Table 1) [7-16]. CMV appendicitis typically presents with similar symptoms [17,18], but may have a higher mortality than other identified etiologies of appendicitis [12,17].

**Table 1:** CMV appendicitis cases reported in HIV-infected patients from 1988 to present

**Reference/Year**	**Age/Gender**	**ARVs at diagnosis (duration)**	**Clinical syndrome**	**Diagnosis**	**Treatment**	**Outcome**
[6] 1989	35 yo M	No	Abdominal pain, TTP in RLQ, rebound	Histology	Laparoscopic appendectomy with IV and oral antibiotics	Abscess; Recovery then readmission and death 25 days later
[7] 1988 (Published in 1991)	50 yo M	No	Fever, RLQ pain, rebound tenderness, RLQ mass	Histology	Exploratory laparotomy; IV antibiotics ganciclovir 5×/week (“maintenance therapy”) when CMV confirmed	Periappendiceal abscess; Recovery
[8] 1990	28 yo M	Yes (5 weeks)	RLQ pain, rebound	Histology	Exploratory laparotomy, IV antibiotics with appendectomy	Recovery
[9] 1991	31 yo M	No	Fever, RLQ pain, rebound, guarding	Histology, in situ hybridization with CMV probes	Appendectomy	Recovery
[10] 1993	38 yo M	NR	RLQ pain, fever TTP at McBurney’s point	Histology, IHC (monoclonal anti-CMV antibody)	IV antibiotics and observation; Exploratory laparotomy with appendectomy; ganciclovir	Recovery
[11] 1994 Patient 1	48 yo M	NR	Lower abdominal pain, fever, septic shock	Histology	Exploratory laparotomy with appendectomy, ganciclovir, imipenem, inotropes.	Perforation; Death
[11] 1994 Patient 2	27 yo M	NR	Periumbilical pain, nausea, vomiting, peritoneal signs	Histology, IHC	Exploratory laparotomy with appendectomy	Death
[12] 1995	34 yo M	Yes (unknown duration)	RLQ pain, fever	Histology CMV PCR in WBC	Laparoscopic appendectomy	Recovery
[13] 1997	30 yo M	NR	RLQ pain, fever, nausea, vomiting	Histology	Appendectomy, post-op ganciclovir	Recovery
[14] 1997	29 yo M	NR	Right sided abdominal pain	Histology, immunofluorescence	Appendectomy	Abscess and perforation; Recovery
[15] 2004	37 yo M	NR	Fever, abdominal pain, rebound and guarding over RLQ	Histology, immunostaining	Appendectomy; IV ganciclovir	Recovery
Present case	38 yo F	Yes (2 weeks)	RLQ pain, anorexia, nausea, fever	Histology, CMV viral staining	Laparoscopic appendectomy, IV antibiotics peri-operatively, valganciclovir (2 weeks)	Perforation; Full recovery

NR: not reported; M: male; F: female; RLQ: right lower quadrant; TTP: tenderness to palpation, IHC: immunohistochemistry. WBC: white blood cells.

In a study of autopsies of HIV positive patients, CMV was the most commonly isolated opportunistic infection, though the vast majority of the patients were asymptomatic while alive [19]. In this particular patient, the biopsy demonstrated substantial inflammation and necrosis as well as abundant CMV positive cells, making CMV the likely cause of appendicitis, rather than an incidental finding.

## Case presentation

The patient enrolled in an Institutional Review Board-approved, prospective study of HIV-1 infected, ART naïve patients with CD4 count below 100 cells/μL in Bethesda, Maryland, one month after a new HIV diagnosis. At the time of enrollment, the patient complained of a 23 Kg weight loss and had a CD4 count of 72 T cells/μL and a plasma HIV-RNA of 284,010 copies/ml. The viral genotype showed wild type virus and she was initiated on Efavirenz/Emtricitabine/Tenofovir. Her blood CMV PCR was negative at baseline, CMV IgG was positive (4.460 U) and CMV IgM was negative.

Two weeks after ART initiation, the patient returned to care with a four-day history of cramping abdominal pain predominantly in the right lower quadrant as well as nausea and anorexia without chills, vomiting, diarrhea, urinary symptoms, or vaginal discharge. The patient was febrile (38.8°C) and tachycardic (124 bpm). Abdominal exam revealed normoactive bowel sounds with direct tenderness to palpation in the lower abdominal quadrants (right more than left). Guarding, rigidity, and rebound were absent and the remainder of the exam was noncontributory.

**Figure 1:**
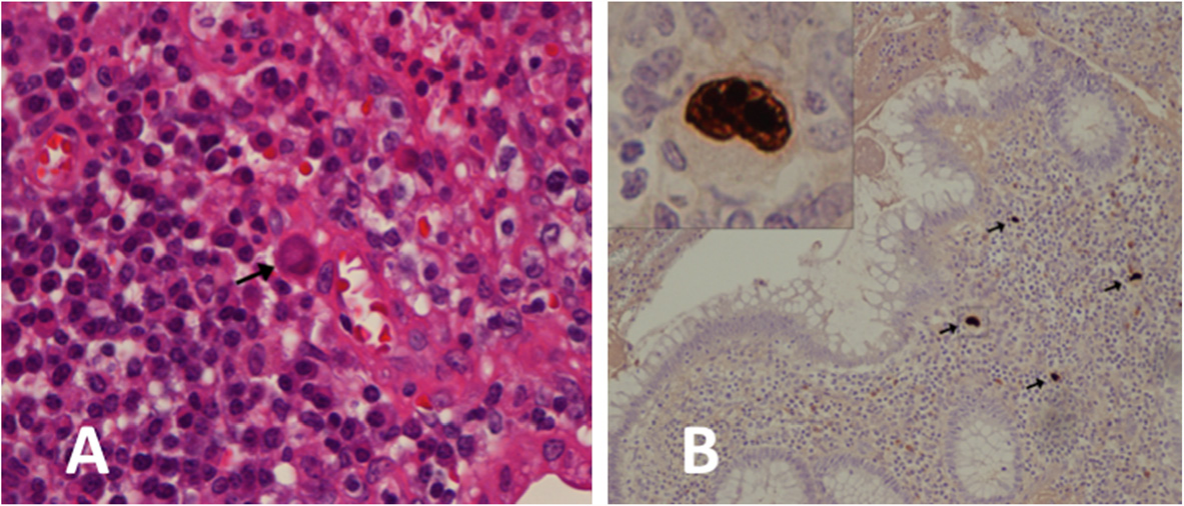
**Histopathologic examination of the appendix. A**: Hematoxylin and eosin stain showing lymphoplasmacytic hyperplasia and scattered enlarged cells (arrow) with features characteristic of CMV-induced cytopathic changes. **B**: CMV immunostaining demonstrating CMV-infected cells throughout the full thickness of the bowel wall (arrows). Figure inset illustrates an infected cell at higher power view depicting cellular and nuclear enlargement with nuclear inclusions.

CBC demonstrated leukopenia (leukocytes 2.58 K/μL) with 0.8% immature granulocytes. Alkaline phosphatase was 186 IU/L (up from 140 IU/L at baseline), with normal liver and pancreatic enzyme levels. The CRP had increased to 8.52 mg/dl from 1.95 mg/dl at baseline. Blood CMV PCR became detectable at 750 copies/ml. An abdominal CT scan demonstrated thickening of the appendix with fat stranding and mild lymphadenopathy of the pelvic sidewall, predominantly on the right side consistent with appendicitis. The patient underwent an uncomplicated laparoscopic appendectomy, received IV metronidazole and vancomycin perioperatively, and was discharged on post-op day three.Pathologic examination of the patient’s appendiceal tissue demonstrated appendicitis with sealed perforation and evidence of CMV infection. There was marked lymphoid hyperplasia with mature lymphocytes and plasma cells (Figure 1A). Immunohistochemistry showed that the lymphocytic infiltration was composed of a mixture of T cells and polyclonal B cells with equal distribution of immunoglobulin light chain kappa and lambda staining, making lymphoma unlikely. Neutrophilic infiltration was scarce. In situ hybridization for EBV virus encoded small RNA (EBER) was negative. CMV viral staining was positive in numerous cells showing typical cytopathic changes of CMV infection including cellular and nuclear enlargement and nuclear inclusions (Figure 1B), and were distributed throughout the full thickness of the bowel wall.

The patient returned to clinic after 3 weeks with complaints of increased abdominal pain and two episodes of hematochezia. Valganciclovir was initiated for suspected CMV colitis (endoscopy could not be performed due to the recent abdominal surgery). The patient completed two weeks of valganciclovir therapy with complete resolution of all symptoms.

## Conclusions

It has been hypothesized that appendicitis in HIV positive patients could be a result of IRIS, likely due to reactive lymphoid tissue in the appendix [20,21]. Although CMV-IRIS in HIV-infected patients has been mostly reported as uveitis in those with CMV retinitis [22], appendicitis can be a rare manifestation of CMV in this clinical setting. This patient represents the first published case of appendicitis as a result of CMV-IRIS. Given the frequency of CMV co-infection and wide distribution of CMV in the gastrointestinal tracts of patients with AIDS, a proportion of excess cases of appendicitis in the HIV-infected population may be related to emergent immune responses to CMV after ART initiation.

## Consent

Written informed consent was obtained from the patient for publication of this case report and any accompanying images. A copy of the written consent is available for review by the Editor of this journal.

## Competing interests

The authors declare that they have no competing interests.

## Authors’ contributions

VS and SK provided clinical care for the patient. HWW provided the pathological diagnosis and images. KF, VS, and IS wrote the manuscript. All others read and approved the final manuscript.

## Pre-publication history

The pre-publication history for this paper can be accessed here:

http://www.biomedcentral.com/1471-2334/14/313/prepub
